# Winter Cough

**Published:** 1890-06

**Authors:** Frederic Preston

**Affiliations:** Chester, Pa.


### WINTER COUGH.

FREDERIC PRESTON, M. D., CHESTER, PA.

Coming on regularly for twelve successive winters. Tall, spare man of seventy-three years. Cough constant during night and early morning. The victim had been obliged to pass his winter nights sitting by stool; lying impossible. Many nostrums and some homœopathic remedies tried, among others Hyosc. The cough was without sputum, except when outdoors. He had contracted the habit of going out into the cold air several times nightly during cold season to cough up a small portion of white, glairy mucus, which gave from one to three hours' relief. Three doses of Plantago major$^{200}$ cured this cough within twenty-four hours. Since January 12th, 1887, no return. See Allen, Vol. VII, p. 564. Plantago.

